# Implementation rate and effects of multidisciplinary team meetings on decision making about radiotherapy: an observational study at a single Japanese institution

**DOI:** 10.1186/s12911-022-01849-y

**Published:** 2022-04-27

**Authors:** Mayumi Ichikawa, Ken Uematsu, Natsuko Yano, Masayoshi Yamada, Takashi Ono, Shohei Kawashiro, Hiroko Akamatsu, Yasuhito Hagiwara, Hiraku Sato, Kenji Nemoto

**Affiliations:** Department of Radiation Oncology, Yamagata University School of Medicine, 2-2-2, Iida-nishi, Yamagata, 990-9585 Japan

**Keywords:** Multidisciplinary team meetings, Tumor board, Cancer, Oncology, Radiotherapy, Decision making

## Abstract

**Background:**

Cancer treatment requires a multidisciplinary approach. Therefore, multidisciplinary team meetings (MDTMs) have been widely used to determine the direction of treatment. However, no standard provisions exist for conducting MDTMs, and recommendations discussed in MDTMs are sometimes not implemented. ​This study analyzed the indications for radiotherapy discussed and recommended at MDTMs, identified the rate of radiotherapy recommendations for patients that were not implemented, and clarified the reasons at a single academic center in Japan.

**Methods:**

This was a cross-sectional study that analyzed the minutes and electronic medical records of cases discussed at MDTMs held between April 2012-March 2017 at Yamagata University Hospital. We categorized how radiotherapy was initially presented at MDTMs, determined the rate of radiotherapy recommendations made through MDTMs, analyzed whether treatment recommendations were subsequently implemented, and examined the causes of non-implementation. We performed a statistical analysis to assess some clinical factors (sex, age, number of multidisciplinary team meetings, and classification of planned treatment) associated with the non-implementation of radiotherapy recommendations from MDTMs.

**Results:**

A total of 1813 cases were discussed at MDTMs, of which 71% (1293 cases) were presented with treatment plans, including radiotherapy. Further, 66% (1205 cases) were recommended for radiotherapy through the MDTMs. Recommendations from MDTMs were not implemented in 7% (142 cases). The most typical reason for non-implementation was the clinician’s opinion (30%), followed by patient preferences (27%) and disease progression (20%). Change in cancer stage and improvement in symptoms were 12% and 4%, respectively. These ratios were similar each year. We could not find the factors associated with the non-implementation of radiotherapy recommendations from MDTMs.

**Conclusions:**

MDTMs had a significant effect on the recommendation of radiotherapy for each patient with a tumor. The primary reason for the non-implementation of decisions made at MDTMs was the opinion of clinicians and the patient’s preference. These results were similar to previous studies. We need to establish a monitoring system where patients themselves can decide the treatments based on their choices while using the recommendations from MDTMs.

## Background

Multidisciplinary treatment is necessary for patients with cancer, for whom a cross-sectional therapeutic and care approach is required. In 1995, the UK Department of Health framework identified the multidisciplinary treatment team as a component of the multidisciplinary treatment approach. The team comprises surgeons, physicians, radiologists, radiation oncologists, oncologists, pathologists, palliative care physicians, and certified nurses involved in diagnosing and treating patients with cancer. This practice was later adopted in the United States and European countries, and the benefits of multidisciplinary treatment teams have been reported [1–4].

In Japan, the Cancer Control Act, like the UK framework, was enacted by the Ministry of Health, Labor and Welfare in 2008 in response to the demand for appropriate medical care according to the condition of patients with cancer. Specifically, the Act defines multidisciplinary team meetings (MDTMs) as conferences for exchanging opinions, sharing, reviewing, and confirming the symptoms, conditions, and treatment plans for patients with cancer. Such meetings must be held in cancer hospitals at least once per month.

In previous studies from our group, we found that MDTMs resulted in changes in treatment modalities and had an impact on treatment choices such as radiotherapy and chemotherapy [5–7]. Previous studies in other countries have reported on other studies examining the factors necessary to ensure the quality of MDTMs and the factors that influence MDTMs [8–10]. In addition, there are several reports on the non-implementation rate of MDTM recommendations, which generally ranges from 7.8–8.7% [11–13]. However, there are no studies from Japan that have examined the viability of treatment recommendations from MDTMs or what the causes of non-implementation might be. Therefore, this study aimed to analyze how the indications for radiotherapy were discussed and recommended, identified the percentage of patients for whom the recommended radiotherapy was not performed, and clarified the reasons for non-implementation with a focus on radiotherapy recommendations from MDTMs at a single university hospital in Japan.

## Methods

**Fig. 1:**
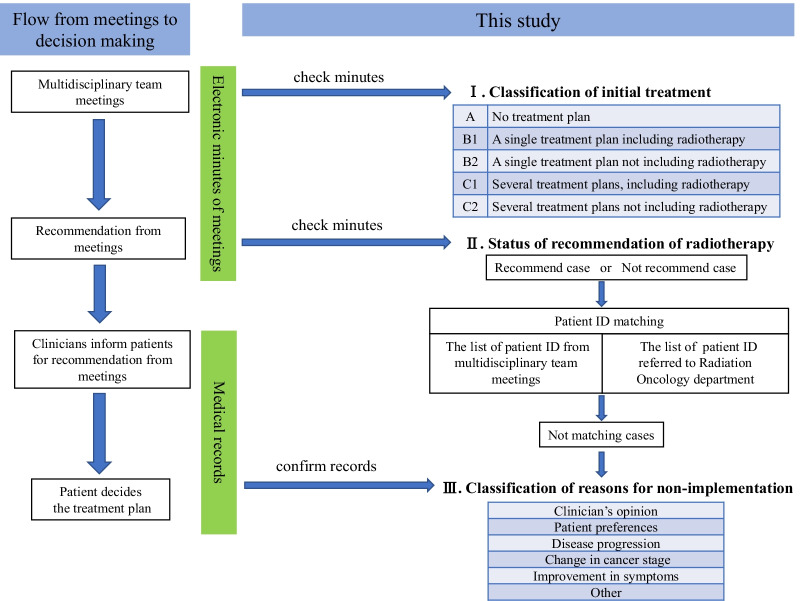
The flow of decision making through meetings and the flow of analysis in this study

This cross-sectional study analyzed the minutes and electronic medical records of cases discussed at MDTMs held between April 2012-March 2017 at Yamagata University Hospital in Japan. MDTMs have been conducted once or twice a week since September 2008 at this regional cancer hospital, with professionals across 13 specialty fields (Hematology, Gastrointestinal, Head and Neck, Breast, Hepatobiliary, Ophthalmology, Bone and Soft Tissue, Lung, Brain, Urology, Pediatric, Gynecology, and Dermatology) discussing approximately 360 cases each year. The core attendees at MDTMs are medical oncologists, radiation oncologists, palliative care specialists, and certified nurses. Radiologists, pharmacists, and pathologists participate as needed. In the MDTMs, most cases are already diagnosed by imaging and pathological methods, and the focus of the discussion is on the best treatment strategy for each case. The case presentation is performed by clinicians (attending physicians or attending surgeons) who may or may not have a treatment plan. The content of the meeting is recorded by the senior resident using the minutes of the meeting, created internally by the hospital's list with Excel. We show the flow of decision-making through MDTMs on the left side of Fig. 1. The attending physician recorded the results of MDTMs’ recommendations in the electronic medical record; after MDTMs, the treatment recommendations are explained to the patient, and finally, the patient decides on the treatment plan. This process is also documented in the electronic medical record by clinicians.

To determine the implementation rate and impact of recommendations made at MDTMs on radiotherapy, this study used minutes and electronic medical records to analyze: (1) how radiotherapy was discussed at MDTMs, (2) whether radiotherapy was recommended throughout MDTMs, and (3) whether the recommendations made at MDTMs were subsequently implemented and the reasons for cases where they were not implemented (see right side of Fig. 1). Moreover, we also investigated whether these results differed from year to year.

First, we classified the cases into five categories, according to the status of initial treatment proposals at MDTMs (Table 1). Category A cases are cases presented without a treatment plan (e.g., *How could we treat this patient?*). Category B1 cases are cases presented with a single treatment plan, including radiotherapy (e.g., *What about radiotherapy alone?*). Category B2 cases are those presented with a single treatment plan, not including radiotherapy (e.g., *What about surgery alone? Or, chemotherapy alone? Or, best supportive care alone?*). Category C1 cases are cases presented with several treatment plans, including radiotherapy (e.g., *Which would be better, chemoradiotherapy or surgery?*). Category C2 cases are cases that are presented with several treatment plans, not including radiotherapy (e.g., *Which would be better, chemotherapy or surgery?*).

**Table 1:** Classification of initial treatment proposals presented at MDTMs*

A	No treatment plan
B1	A single treatment plan including radiotherapy
B2	A single treatment plan not including radiotherapy
C1	Several treatment plans, including radiotherapy
C2	Several treatment plans not including radiotherapy

*Multidisciplinary team meetings

Next, we examined how the recommendations for radiotherapy changed with MDTMs compared with the status of the initial treatment proposals (Fig. 1). The method for this involved confirming whether the cases judged to be suitable for radiation therapy by the meeting had radiotherapy later by matching the patient ID list in the MDTMs’ minutes with the ID list of new patients for radiation therapy within the next few months. Then, we classified them as meeting recommendation implemented or non-implemented cases. Subsequently, we manually categorized the reasons for non-implemented cases based on the descriptions in the medical records (Fig. 1).

Statistical analysis was performed using SPSS Version 23 (IBM: Armonk, NY, USA). We conducted a univariate analysis with some clinical variables (sex, age, number of multidisciplinary team meetings, and classification of planned treatment) to determine whether clinical factors affected the implementation of radiotherapy recommendations from MDTMs. The data were stratified as follows: for age, we set it at under 70 years or over; for the number of MDTMs, we stratified in the initial or later MDTMs; for pre-treatment options, we stratified category A or another.

## Results

There were 1813 cases discussed at MDTMs between April 2012-March 2017. This included 1463 patients; 1111 patients were discussed once, 184 patients were discussed twice, 64 patients were discussed 3 times, 23 patients were discussed 4 times, 6 patients were discussed 5 times, 2 patients were discussed 6 times, and 1 patient was discussed 8 times.

The case characteristics are shown in Table 2. The overall median age of patients was 71 years. The most common specialty fields were Lung, Urology, and Brain. Ophthalmology and Dermatology, which are less common, had fewer cases. Breast, which should have a large number of cases, had very few cases (1.2%).

**Table 2:** Cases characteristics

Number of cases		n = 1813	
Sex	Male/female	1173/640	
Age in years	Range (mean)	0–95 (71)	
Specialty fields	Lung	421	23.2%
	Urology	387	21.3%
	Brain	222	12.2%
	Hematology	165	9.1%
	Gastrointestinal	162	8.9%
	Gynecology	114	6.3%
	Head and neck	99	5.5%
	Hepatobiliary	67	3.7%
	Bone and soft tissue	66	3.6%
	Pediatric	37	2.0%
	Ophthalmology	26	1.4%
	Breast	21	1.2%
	Dermatology	20	1.1%
	Unknown	6	0.3%

The number of patients in each planned treatment classification is shown in Table 3. Category B1 was the most common, with 1080 cases. Category A of all cases was 224 cases, category B2 was 260 cases, category C1 was 213 cases, and category C2 was 36 cases when the initial treatment proposals were presented at MDTMs. As a result, 71% (category B1 + category C1: 1293 cases) were presented with treatment plans, including radiotherapy.

**Table 3:** Percentage of recommendations for radiotherapy and implementation of MDTMs’^*^ decisions

Pre-meetings		Post-meetings (pre-decision making)				Post-decision making			
Classification of initial treatment proposals	Number of cases	Radiotherapy				Decision of meetings			
		Recommended cases		Not recommended		Implemented		Not implemented	
Total	1813	1205		608		1689	93%	124	7%
A	224	81	36%	143	64%	209		15	
B1	1080	982	91%	98	9%	1014		66	
B2	260	14	5%	246	95%	250		10	
C1	213	122	57%	91	43%	182		31	
C2	36	6	17%	30	83%	34		2	

*Multidisciplinary team meetings

After the time of the presentation to the meeting, radiotherapy was recommended in 66% (n = 1205) of all cases as the result of MDTMs. Of category B1 cases, for whom radiation therapy was planned, radiotherapy was not selected as a recommended treatment in 9% (n = 98). Of category B2 cases, for whom radiation therapy was not planned, radiotherapy was recommended in 5% (n = 14). Of category C1 cases, for whom radiation therapy was one of the treatment options, radiation therapy was not recommended in 43% (n = 91). Of category C2 cases, for whom radiation therapy was excluded as a treatment option, radiation therapy was recommended in 17% (n = 6). The implementation rate of radiotherapy with patient ID matching confirmation was high (n = 1689, 93%), but 7% (124 cases) were not implemented.

We show reasons for non-implementation in Table 4. The most common reason for non-implementation was clinician’s opinion (30%, n = 37), followed by patient preferences (27%, n = 34), and disease progression (20%, n = 25). Change in cancer stage and improvement in symptoms were 12% (n = 15) and 4% (n = 5), respectively.

**Table 4:** Reasons for non-implementation

Reasons	n = 124	%
Clinician’s opinion	37	30
Patient preferences	34	27
Disease progression	25	20
Change in stage	15	12
Improvement in symptoms	5	4
Other	8	6

**Fig. 2:**
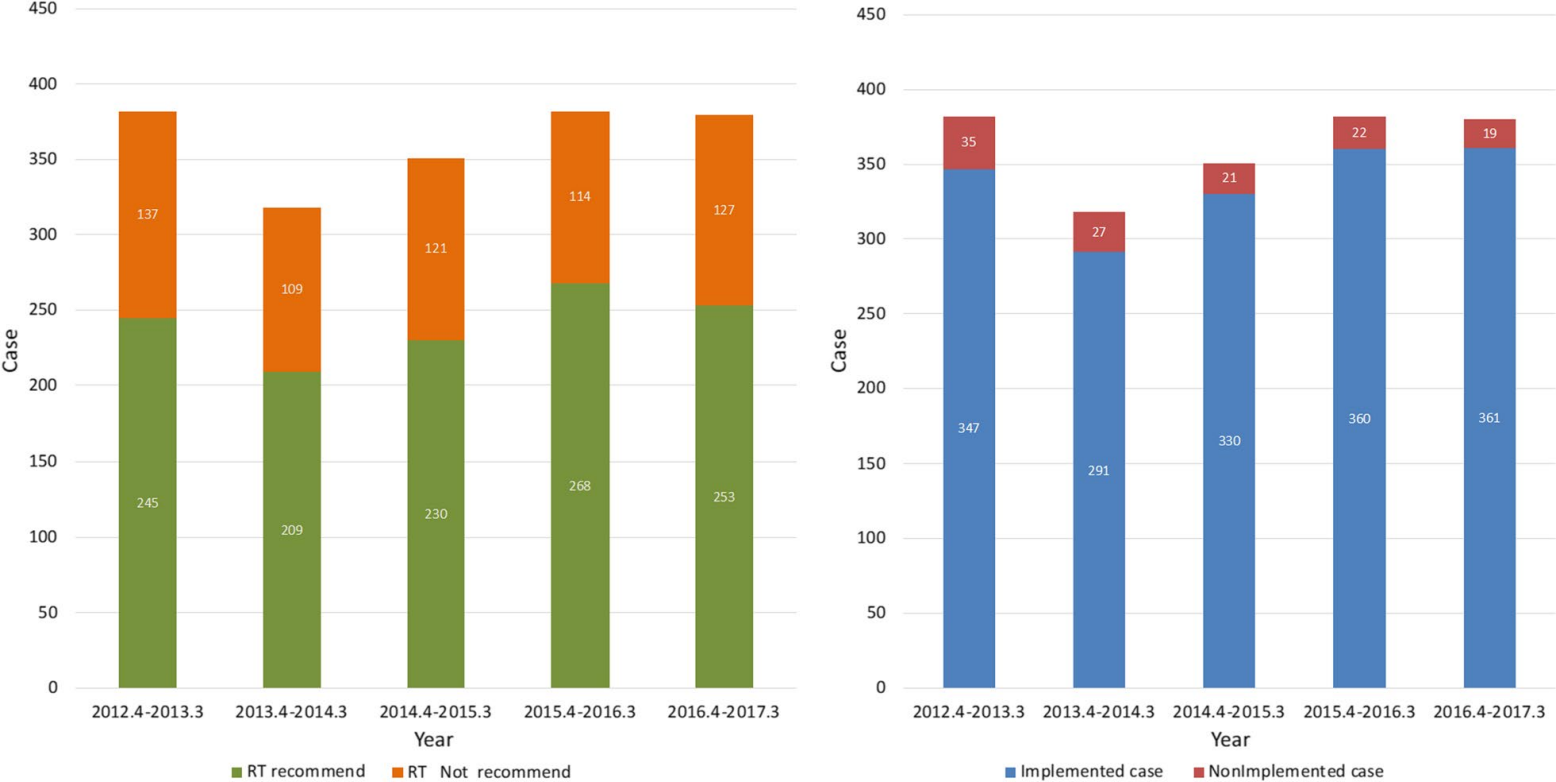
The number of radiotherapy recommendations made and the number of MDTM recommendations not implemented by year

These results were similar from year to year in terms of the percentage of radiotherapy recommendations and the rate of implementation of those recommendations (Fig. 2).

We conducted univariate analysis to determine whether each factor had an effect on the non-implementation rate. It was found that no factor showed a significant difference (sex, p = 0.187; age, p = 0.64; number of MDTMs, p = 0.365; classification of planned treatment, p = 0.078).

## Discussion

In this study, we analyzed how the indications for radiotherapy were discussed and recommended in MDTMs and revealed the non-implementation rate and the causes of non-implementation. We found that the recommended rate of radiotherapy in MDTMs was high. Previous reports have also shown the effect of MDTMs on radiotherapy. Lan et al. showed a significant increase in the use of radiotherapy in colorectal cancer patients by MDTMs [14]. Similarly, Boxer et al. showed that MDTMs lead to a significant increase in the use of radiotherapy in lung cancer [15]. Similarly, the influence of MDTMs on treatment decisions has been reported for various cancers. Forrest et al. reported that the rate of palliative care decreased, and the rate of active chemotherapy increased after MDTMs were introduced in inoperable patients with non-small cell lung cancer [16]. Schmidt et al. reported that MDTMs caused recommendations to differ from the administrator's initial plan in 26–40% of cases of lung cancer patients, and Ung et al. analyzed that MDTMs changed management plans in 58% of the cases [17, 18]. For gynecologic tumors, Gatcliffe et al. prospectively surveyed MDTMs and reported patient assessment or management changes in 34.6% of patients, with significant changes found in 24.5% of the cases [19]. ﻿Cohen et al. assessed the role of MDTMs in the management of gynecologic cancers and found major (5.9%) and minor (3.1%) changes in 9% of patients and standard changes in patient management that resulted from MDTMs with the addition of chemotherapy and surgery [20]. For breast cancer, Murthy et al. reported that 42.1% of the patients had changed management plans through MDTMs [21]. Pawlik et al. evaluated the impact of MDTMs on the management of pancreatic cancer and found that 38 of 203 (18.7%) patients had a change in the status of their clinical stage after a review of submitted imaging [22]. De Luca et al. indicated that the uro-oncology MDTMs alter management plans in at least one-quarter of patients, reaching almost 50% of cases in locally advanced disease [23]. Thus, previous studies have shown that MDTMs contribute to deciding the treatment direction.

However, in our study, 7% of the treatment directions recommended about radiotherapy at the MDTMs were not implemented. The most common reason for non-implementation was clinician’s opinion (30%, n = 37), followed by patient preferences (27%, n = 34). This non-implementation rate and the primary reasons were similar to other multiple MDTM studies, with clinician’s decision at 23–24% and patient preference at 28–36% [12, 13]. The results for the reason for clinician’s opinion may be explained by the difference in views between clinicians and radiation oncologists. Fowler et al. in a comparison of recommendations by urologists and radiation oncologists for the treatment of clinically localized prostate cancer, reported that 93% of urologists tend to recommend radical prostatectomy, whereas 72% of radiation oncologists believe that surgery and external beam radiotherapy are equivalent treatments [24]. Furthermore, Ariane et al. revealed significant differences in therapeutic approaches between urologists and radiation oncologists who deal with localized prostate cancer [25]. For intermediate-risk prostate cancer in a 65-year-old patient, 96.5% of urologists chose radical prostatectomy versus 37.7% of radiation oncologists.

Meanwhile, recommendations at MDTMs may not be implemented for other reasons. Blazeby et al. investigated the implementation of such decisions in upper-gastrointestinal cancer cases and found discordance in 15.1% of the cases, with the central reasons for discordance being comorbid health issues (43.9%), patient choice (34.2%), and decision changes when more clinical information becomes available (19.5%) [26]. In a similar study of breast cancer cases in the United Kingdom, English et al. identified the most common reason for discordance as patient preferences (65%); other reasons are the discovery of new clinical information and surgeons’ views [27].

In this study, we could not find a factor of non-implementation within the statistics for patients who were not treated according to the recommendation at MDTMs. However, previous studies revealed that factors of non-implementation were tumor site and comorbidities [11, 13, 28].

The number of non-implemented cases tended to be higher among cases in category C1 (multiple treatment plans including radiotherapy as the initial treatment plan), and the proportion of cases in hematological and gastrointestinal diseases tended to be higher than in other fields (Table 5). Hematological cases may be more prone to changes in symptoms than cases in other fields, and the percentage of non-implemented cases may be higher. It is unclear why there were many non-implemented cases for cases of gastrointestinal cancer, but it may be that fewer cases are presenting to MDTMs, and low activity against MDTMs tends to result in more non-implemented cases.

**Table 5:** Details on the non-implementation of MDTMs’** decisions

Non-implemented decision		n = 124	%*
Classification of initial treatment proposals	A	15	6.7
	B1	66	6.1
	B2	10	3.8
	C1	31	14.6
	C2	2	5.6
Specialty fields	Hematology	22	26.8
	Gastrointestinal	25	15.4
	Head and neck	10	10.1
	Breast	2	9.5
	Hepatobiliary	5	8.1
	Ophthalmology	2	7.7
	Bone and soft tissue	5	7.6
	Lung	31	7.4
	Brain	8	3.6
	Urology	12	3.1
	Pediatric	1	2.7
	Gynecology	1	0.9
	Dermatology	0	0.0
	Unknown	0	0.0
Recommendation of radiotherapy at meetings	Recommend	102	8.5
	Not recommend	22	3.6

*Rates of all cases for each item

**Multidisciplinary team meetings

This study has several limitations. This is a retrospective study at a single institution in Japan, and the case mix is not the typical proportion of cancer cases in Japan, and hence, may have a bias in the number of cases. In addition, the confirmation of the reason for not conducting the study is not a fixed item extraction but a manual confirmation from the medical record description. Furthermore, the failure to identify influencing factors was most likely due to insufficient clinical variables.

We will continue to check the consistency of the treatment recommended by MDTMs, but we need to establish a monitoring system to ensure that recommended treatments are adequately explained by clinicians and decision-making is based on patients’ choices.

## Conclusions

The rate of radiotherapy recommendations at MDTMs was high, and the implementation rate after MDTMs was also high. Therefore, we concluded that MDTMs led to a significant effect on the recommendation of radiotherapy and were effective in determining the treatment plan for each patient with a tumor.

The primary reason for the non-implementation of decisions made in MDTMs was the opinion of clinicians and the patient's preference, and these results were similar to previous studies. We need to establish a system where patients can monitor the treatments they receive based on their choices.

## Data Availability

We will allow the journal to review our data if requested. All raw data are written in Japanese. The data material used in this study is available from the corresponding author on reasonable request, provided it does not conflict with the anonymity and confidentiality of the data.

## Abbreviation

MDTMs: Multidisciplinary team meetings
